# Differences in the Pattern of Non-Recreational Sharing of Prescription Analgesics among Patients in Rural and Urban Areas

**DOI:** 10.3390/healthcare9050541

**Published:** 2021-05-06

**Authors:** Filipa Markotic, Mario Curkovic, Tanja Pekez-Pavlisko, Davorka Vrdoljak, Zeljko Vojvodic, Dinka Jurisic, Marijana Puljiz, Martina Novinscak, Karmela Bonassin, Snjezana Permozer Hajdarovic, Marion Tomicic, Ines Diminic-Lisica, Sonja Fabris Ivsic, Danijel Nejasmic, Ivana Miosic, Ivana Novak, Livia Puljak

**Affiliations:** 1 Department for Assessment of Safety and Efficacy, Croatian Agency for Medicinal Products and Medical Devices, 10000 Zagreb, Croatia; 2 Department of Family Medicine, School of Medicine, Josip Juraj Strossmayer University of Osijek, 31000 Osijek, Croatia; mcurkov@yahoo.com; 3 Department of Family Medicine, Health Centre Kutina, 44320 Kutina, Croatia; tashamed@gmail.com; 4 Department of Family Medicine, School of Medicine, University of Split, 21000 Split, Croatia; marion.tomicic@mefst.hr; 5 Department of Family Medicine, Health Centre Osijek, 31204 Bijelo Brdo, Croatia; vojvodic58@gmail.com; 6 Department of Family Medicine, Health Centre Sisak, 44272 Lekenik, Croatia; dinka.jurisic@hotmail.com; 7 Department of Family Medicine, Health Centre Imotski, 21262 Kamenmost, Croatia; marijanapuljiz@gmail.com; 8 Department of Family Medicine, Health Centre Cakovec, 40000 Cakovec, Croatia; mnovinscak@gmail.com; 9 Department of Family Medicine, Istrian Health Centre, 52341 Zminj, Croatia; karmelabonassin@gmail.com (K.B.); sonja.fabris.ivsic@idz.hr (S.F.I.); 10 Department of Family Medicine, Health Centre Cakovec, 40329 Kotoriba, Croatia; snjezanaph@gmail.com; 11 Department of Family Medicine, Split-Dalmatia Health Center, 21000 Split, Croatia; 12 Department of Family Medicine, School of Medicine, University of Rijeka, 51221 Kostrena, Croatia; ines.diminic.lisica@medri.uniri.hr; 13 Department of Physics, School of Medicine, University of Split, 21000 Split, Croatia; dnejasmic@gmail.com; 14 Center for Evidence-Based Medicine and Health Care, Catholic University of Croatia, 10000 Zagreb, Croatia; ivanamiosic1@gmail.com (I.M.); ivana.i.novak@gmail.com (I.N.); livia.puljak@unicath.hr (L.P.)

**Keywords:** prescription analgesic, sharing, lending, borrowing, rural, urban

## Abstract

Introduction: This study aimed to analyze differences in sharing of prescription analgesics between rural and urban populations. Methods: We surveyed 1000 participants in outpatient family medicine settings in Croatia. We used a 35-item questionnaire to analyze patients’ characteristics, pain intensity, prescription analgesic sharing behavior, and perception of risks regarding sharing prescription medications. Results: Prescription analgesic sharing was significantly more frequent in the rural (64%) than in the urban population 55% (*p* = 0.01). Participants from rural areas more commonly asked for verbal or written information than those from urban areas when taking others’ prescription analgesics (*p* < 0.001) or giving such analgesics (*p* < 0.001). Participants from rural areas more commonly informed their physician about such behavior compared to those from urban areas (*p* < 0.01), and they were significantly more often asked about such behavior by their physician (p < 0.01). Perceptions about risks associated with sharing prescription medication were similar between rural and urban populations. Conclusions: There are systematic differences in the frequency of prescription analgesics and associated behaviors between patients in family medicine who live in rural and urban areas. Patients from rural areas were more prone to share prescription analgesics. Future studies should examine reasons for differences in sharing prescription analgesics between rural and urban areas.

## 1. Introduction

Sharing a prescription medication with another person to whom the prescription was not prescribed is common behavior, with prevalence ranging from 5% to 61% [1,2,3,4,5,6,7,8,9,10,11,12,13,14,15]. Patients share their prescription medications by giving (lending) their medications to another person or taking (borrowing) medications from another person who is not a physician [1]. Medications are shared for recreational (non-medical) or non-recreational (medical) purposes. Non-recreational sharing of prescription medication is more frequent than recreational sharing [16].

It has been described that such behaviors could have both negative and positive aspects. Negative aspects may include adverse health consequences associated with consummation of an incorrect drug, greater risk of developing resistance to drugs, increased risk of adverse effects, or delayed seeking of medical care [8,17,18]. Described positive aspects associated with sharing of prescription medications may include the availability of a medication when medical aid is unavailable, or it is inconvenient to contact a physician, saving money, helping patients cope with their medical problems, accessibility of drugs in pharmacies, availability of medication provided by close individuals and maintaining good interpersonal relationships [17,19,20].

It has been reported that chronic pain has a high prevalence (23%) in Spain’s population and was more prevalent amongst women (31%), older people (40%), work-disabled persons (55%), housewives (36%), retired persons (34%) and farmers (33%) [21].

Analgesics belong to a group of prescription medications that are most commonly shared among patients [6,7,9,11]. The main reasons why prescription analgesics are shared include reducing the suffering in people from their community and keeping good interpersonal relationships with them [20]. We were drawn to this topic while conducting a study about adherence to prescription analgesics among elderly persons. In that study, which was published in 2013, 28% of participants indicated that they shared their prescription analgesics with other persons, including family, neighbors, and friends [10].

In 2014, Beyene et al. published a systematic review in which they highlighted a knowledge gap about the perception of risk on this topic and warned that it is not known whether patients perceived sharing of medications as a potentially risky behavior [11]. In our earlier qualitative study, we found that patients usually were aware of the risks this behavior may cause, but they considered that potential benefits outweigh risks. Furthermore, we found that patients do not recognize the risks associated with sharing prescription analgesics [20]. Keeping leftover medicines at home was found to be a positive predictor of future sharing of medicine [22]. Our study among pain management physicians indicated that sharing of prescription analgesics is a neglected problem that requires more attention by physicians [19].

Croatia has a high standard of health care in the overall population. According to the data provided by the Croatian Health Insurance Fund (CHIF), almost the entire population, with over 4.1 million inhabitants, has access to full-range health care [23]. This means that insured persons are entitled to all medications and tests on the list of the CHIF at no additional cost or with a minimal supplementary payment. The list of CHIF-funded analgesics contains almost all nonsteroidal anti-inflammatory drugs (NSAIDs), tramadol, tramadol in combination with paracetamol, morphine, oxycodone, tapentadol, fentanyl, and buprenorphine patch [24]. All opioid analgesics in Croatia, apart from tramadol, have been under strict prescription control and can be prescribed for an individual patient by one family physician under double-checking arrangements, both in the clinic and the pharmacy. Only a few NSAIDs and paracetamol can be purchased as over-the-counter (OTC) medicines in Croatia, and only in pharmacies and specialized shops, but not in other types of stores, such as supermarkets. In terms of overall consumption, the ratio between the medications from the CHIF list and OTC medications is 91.3 to 8.7 percent in favor of the former [25].

Various determinants may influence patients’ health-related behavior regarding sharing of prescription analgesics, including rural/urban dwellings. Identifying determinants associated with undesirable health behaviors can help design interventions to reduce such behaviors. The previous literature has not described whether there are differences in the sharing of analgesics between the rural and urban populations. Rural physician–patient interactions tended to be more focused on socioemotional communication and relationship-building than the urban interactions [26]. Also, the urban population may have fewer difficulties accessing pharmacy than the rural population [27]. According to the previous research in Croatia, the rural population more frequently reported poor health and difficulties accessing health services than the urban population. However, the rural population expressed more trust in the healthcare system than the urban population [28].

According to the Croatian Bureau of Statistics, based on the census from 2011, there were 2,324,144 inhabitants living in cities, making up 54.2% of the total number of inhabitants in Croatia [29]. Thus, almost half of Croatian inhabitants live in villages, making it important to study differences between urban and rural populations.

Thus, this study aimed to analyze differences in the pattern of non-recreational sharing of prescription analgesics between rural and urban populations.

## 2. Materials and Methods

### 2.1. Study Design

We conducted a cross-sectional study among the convenience sample of 1000 adult participants (500 participants from rural and 500 participants from the urban area) to whom their physicians have prescribed analgesics at least once in their lifetime. This is a secondary analysis of a large study we conducted in this setting [15]. The Ethics Committee of the University of Split School of Medicine approved the study protocol. All participants gave verbal and written consent to participate in the study.

### 2.2. Setting

The study included consecutive participants from 10 outpatient family medicine practices in 5 Croatian regions: central Croatia, eastern Croatia, northwest Croatia, northern Adriatic and Lika, central and southern Adriatic. Urban places where the study was conducted were Osijek, Split, Cakovec, Kutina, Rijeka; rural places were Lekenik, Zminj, Kamenmost, Bijelo Brdo, Kotoriba.

Two practices were included in every region, one in an urban and one in a rural area per 100 participants.

Participants were recruited when they visited a family medicine practice. Consecutive eligible participants got an invitation to participate in the research and detailed data about the research via a family physician who enrolled them. The surveys were conducted by family physician from 25 January 2016 to 30 June 2016.

### 2.3. Inclusion and Exclusion Criteria

Inclusion criteria were: adults aged ≥ 18 years who had received prescription analgesics at least once in their lifetime. Exclusion criteria were: cognitive disorders (e.g., dementia) and mental illness (e.g., uncontrolled schizophrenia), which would prevent understanding the questionnaire. Participants were consecutively included as they visited the family medicine practice until each practice has included 100 participants.

### 2.4. Questionnaire

Data were collected via a 35-item questionnaire [15]. The questionnaire was piloted among five researchers and five laypersons to ensure that wording, content, and language were appropriate. Feedback from the pilot testing was incorporated in the final version of the questionnaire. In the questionnaire, participants were asked for: (1) a list of analgesics they are currently taking, (2) their beliefs about their prescribed analgesics, (3) duration of pain and pain intensity on the 10-item numerical pain scale (where 1 is no pain and 10 worst possible pain), using a number which best described their pain during the last week, (4) about their lending and/or borrowing of prescription analgesics; respondents were directly asked whether they lent and/or borrowed medication, (5) their perception of risks associated with sharing medications, (6) participants’ behavior regarding sharing prescription analgesics, (7) their personal characteristics, and (8) demographic data. The participants were told to ask their family doctors if they are unsure which of their medications is prescription or not.

### 2.5. Data Collected

The following characteristics of the patients were collected via questionnaire: whether they read the paper-based instructions (package inserts) that come with medication; the tendency of taking (multi) vitamin supplements or other supplements; tendency to search for health information on the Internet; the tendency of respondents using the services of alternative medicine; personal assessment of their medical condition and attitudes towards medications.

The participants’ demographic variables collected were household size, education level, sex, age, region of residence, and residence in an urban or rural area.

### 2.6. Statistics

Frequencies and percentages, medians, and interquartile range (IQR) were used to describe descriptive statistics. Kolmogorov–Smirnov test was used to determine whether data were normally distributed. Chi-square test was used for analyzing differences between rural and urban populations. We conducted analyses using MedCalc v 15.2.1 (MedCalc Software bvba, Ostend, Belgium). Statistical significance was set at *p* < 0.05.

## 3. Results

We included 1000 participants in the study. Among the invited participants, eight patients refused to participate, and two patients who accepted the participation did not return the survey. One of the participants was underage (age < 18 years); we excluded that survey from the analysis and asked a physician to include another participant. There were 32 incomplete surveys. Some of the family physicians collected more than 100 surveys. In total, there were 24 surplus surveys delivered. When needed, we replaced the incomplete survey with the surplus surveys but always kept the number of analyzed surveys from each practice at 100.

Characteristics of the study participants are shown in Table 1. The majority of the participants were women, aged above 50 years, with high school education, suffering from chronic pain lasting more than 3 months. More participants in the rural population suffered from chronic pain than in the urban population. The majority of participants indicated that their analgesics were effective (Table 1).

Participants reported average pain intensity in the past weeks of 4.8 ± 2.5 on the numeric scale 1–10. There was no difference in pain intensity (*p* = 0.096) between urban and rural populations (4.8 ± 2.5 urban vs. 4.8 ± 2.6 rural). On average, at the time of the survey, participants were taking more than one analgesic (1.5 ± 0.7), both in urban (1.4 ± 2.5) and rural (1.6 ± 0.8) populations. The most commonly used non-opioid analgesic was an ibuprofen (*N* = 509; 51%) and among opioids it was a tramadol (*N* = 205; 21%); this was the same in the entire population, as well as in rural (ibuprofen (*N* = 224; 45%) and tramadol (*N* = 102, 20%)) and in urban (ibuprofen (*N* = 285; 57%) and tramadol (*N* = 103, 21%) population.

Overall, 61% (595/975) of the participants engaged in any kind of sharing prescription analgesics’ behavior, i.e., lending and/or borrowing of prescription analgesics. Sharing of prescription analgesics was more frequent in the rural population than in the urban population (*p* = 0.01). The same pattern of persons with whom prescription analgesics were shared was observed in urban and rural populations (Table 2).

Table 3 presents results on participants’ behavior regarding sharing prescription analgesics. Participants from the rural areas significantly less often took verbal or written instructions while taking an analgesic from another person than participants from the urban areas (*p* < 0.001). Likewise, among the participants who were giving their prescription analgesic to other persons, taking verbal or written instructions was more common in the rural population compared to the urban population (*p* < 0.001). Most of the surveyed patients did not inform their physician when they gave their prescription analgesic to someone else. Significantly more participants in rural areas did not inform their physician when they gave their prescription analgesic to someone else, compared to those from urban areas (6.4%) (*p* < 0.01). Most of the participants (*N* = 597, 60%) indicated that their physician never asked whether they gave or took from someone else a prescription analgesic he/she prescribed to them. This was significantly more common among participants from the rural areas (*p* < 0.01).

Participants were then asked to rate the likelihood of various risks associated with sharing prescription analgesics using a 10-item numeric scale (1 = not probable; 10 = very probable). For all suggested risks, the participants had a mean score above 5.0, indicating that, on average, they find these behaviors somewhat or very risky, and there are major differences between participants from rural and urban areas (Table 4).

## 4. Discussion

In this study of 1000 participants from outpatient family medicine, we found systematic differences between participants living in urban and rural areas regarding patterns of prescription-sharing behavior. This behavior was significantly more frequent in the rural population (64%) than in the urban population (55%). Participants from rural areas were more commonly asked for verbal or written information than those from urban areas when taking others’ prescription analgesics or giving such. Participants from rural areas more commonly informed their physician about such behavior than those from rural areas and they were significantly more often asked about such behavior by their physician. Perceptions about risks associated with sharing prescription medication were similar between rural and urban populations.

A study conducted in Nova Scotia in Canada found that residents from urban areas had better access to pharmacies than residents from rural areas [27]. It is likely that the accessibility of pharmacies influences the difference in health-related behavior between urban and rural areas. In our study, there was no difference between rural and urban populations regarding characteristics of persons with whom prescription analgesics were shared. Family members were persons with whom participants shared medications most commonly, as reported elsewhere [9].

Participants from rural areas were more commonly asked for verbal or written information than those from urban areas when taking others’ prescription analgesics or giving such. A previous study conducted in Italy found that the proportion of regular readers of the instruction for medication was somewhat lower among those living in rural areas [30]. The Italian study had a different design; it included 6992 clients of pharmacies, analyzed exposure to two information leaflets, and assessed the degree of acceptability of information presented in the two leaflets. An important finding of that study was that up to 50% of study participants who took over-the-counter medications indicated that they would be willing to change their drug-seeking behavior based on information in the experimental leaflet [30]. This highlights the value of package inserts as information that needs to accompany a drug. Perhaps package inserts should also be used as educational materials for prescription drugs. Such information should be concise, emphasizing what is really of practical importance, and written in a language that patients can easily understand. Thus, patients could be explicitly warned about not giving their medications to other persons to whom they were not prescribed because of potential health-related dangers. Wong et al. have shown no difference in health literacy between the rural and urban populations who take rheumatology drugs. The most disconcerting fact is that up to 15% of rural and urban patients have shown low health literacy and less than one third of patients have incorrectly followed dosing instructions for common rheumatology drugs [31].

In our study, participants from rural areas more commonly informed their physician about such behavior than those from urban areas and they were significantly more often asked about such behavior by their physician. It has already been observed that location of practice, including working in a rural or urban setting, might influence how physicians communicate with patients [26]. A study conducted in rural and urban family practices in Western Canada analyzed communication styles that were used during interactions between physicians and patients. The authors reported that rural and urban physicians spend similar time with their patients, but rural physicians engage their patients in more socioemotional communications, making patient–physician interactions more intrapersonal and this increases patient trust [26].

In our study, perceptions about risks associated with sharing prescription medication were similar between rural and urban populations. A previous study about medication-related behavior such as sharing medication and adherence found that the elderly from urban areas showed better medication-taking behavior [32]. That study was conducted among 401 elderly persons residing in both rural and urban communities. The study found that medication knowledge and behavior were associated with age, gender, education, marital and living status, and health beliefs. The study concluded that education of healthcare workers and the general public regarding medication knowledge and behavior is urgently needed to ensure patient safety [32]. According to the study of Beyene et al., it appears that interventions that take into account health status, psychosocial and behavioral factors have the most success in reducing sharing of medication [33].

We have previously explored independent predictors of prescription analgesic-sharing habits. We found multiple independent predictors of prescription analgesic lending and borrowing, including some nonmodifiable factors, such as younger age, and modifiable factors, such as decreased awareness of personal harm associated with prescription analgesic sharing [15]. Knowledge about these predictors can be used when designing interventions to prevent prescription medication sharing.

In our study, participants from rural areas more frequently suffered from chronic pain, but there was no difference in pain intensity between the areas. On the contrary, a cross-sectional study from Italy found that chronic pain and severe chronic pain were more frequent in the urban than rural population. Similarly, having a rural residence was associated with higher pain grades in Canada [34].

## 5. Limitations

This study had several limitations. Firstly, we used a self-assessment tool to examine the frequency of sharing prescription analgesics, and there may be a degree of unreliability due to reporting bias. Likewise, we used lifetime recall, which may have influenced results, as such questions may be associated with underreporting. Additionally, there is no validated tool analyzing sharing of prescription medications, which is hindering comparative efforts between the studies. Since we used a consecutive sampling of participants who visited their family medicine physician, participants with more interest in health might have prevailed in the sample.

## 6. Conclusions

This study found that living in rural areas is associated with an increased risk for sharing prescription analgesics. Patients from rural areas were more interested in the adequate use of prescription analgesics and more commonly informed their physician about such behavior. There were no differences in the awareness about the risks of sharing prescription analgesics between patients living in rural and urban areas. It seems that patients who live in rural areas have a better relationship and communication with their physicians than patients who live in urban areas. Our results suggest that difficulties in accessing healthcare services could substantially impact medication-sharing behavior. Future studies should examine reasons for differences in sharing prescription analgesics between rural and urban areas.

## Figures and Tables

**Table 1:** Characteristics of the study participants.

Features	Urban (*N* = 500)	Rural (*N* = 500)
Gender, *N* (%)		
Women Men No answer	296 (59.2) 187 (37.4) 16 (3.2)	323 (64.6) 170 (34) 6 (1.2)
Age, years		
Median age IQR No answer	57 18–92 22 (4.4)	51 18–88 13 (2.6)
Number of household members		
Median IQR No answer	3 1–13 18 (3.6)	4 1–8 10 (2)
Education, *N* (%)		
Primary school or less High school College, faculty Master’s, doctorate No answer	66 (13.2) 297 (59.4) 111 (22.2) 9 (1.8) 17 (3.4)	128 (25.6) 307 (61.4) 53 (10.6) 2 (0.4) 10 (2)
Work status, *N* (%)		
Student Unemployed Employed Retired person No answer	11 (2.2) 50 (10) 202 (40.4) 217 (43.4) 20 (4)	4 (0.8) 98 (19.6) 235 (47) 150 (30) 12 (2.4)
Duration of pain, *N* (%)		
<3 months 3 months–5 years ≥5 years No answer	137 (27) 143 (29) 177 (35) 43 (8.6)	104 (21) 163 (33) 195 (39) 38 (7.6)
Opinion regarding prescribed analgesics, *N* (%)		
My drugs are effective My drugs are not effective enough There are too many of them I need more drugs My physician prescribed them too early My physician was supposed to prescribe them sooner No answer	395 (79) 33 (6.6) 16 (3.2) 13 (2.6) 14 (2.8) 6 (1.2) 34 (6.8)	388 (78) 55 (11) 16 (3.2) 35 (7.0) 0 (0) 7 (1.4) 24 (4.8)

IQR = interquartile range.

**Table 2:** Participants’ behavior regarding sharing prescription analgesics.

Features	Urban (*N* = 500)	Rural (*N* = 500)	*p*
Sharing analgesics, *N* (%)			
Lending and/or borrowing Lending Borrowing Lending and borrowing Not sharing No answer	277 (55) 162 (32) 245 (49) 130 (26) 208 (42) 15 (3.0)	318 (64) 253 (51) 292 (58) 227 (45) 172 (34) 10 (2.0)	0.01 <0.00001 0.004 <0.00001 0.01
Lending analgesics with, *N* (%):			
Family Neighbor(s) Extended family Friends and/or acquaintance Colleague Someone else	138 (28) 14 (2.8) 14 (2.8) 17 (3.4) 23 (4.6) 3 (0.6)	210 (42) 50 (10) 44 (8.8) 48 (9.6) 54 (11) 4 (0.8)	0.56 0.002 0.01 0.02 0.07 0.8
Borrowing analgesics with, *N* (%):			
Family Neighbor(s) Extended family Friends and/or acquaintance Colleague Someone else	183 (37) 20 (4.0) 20 (4.0) 34 (6.8) 45 (9.0) 5 (1.0)	235 (47) 40 (8.0) 27 (5.4) 57 (11.4) 65 (13) 10 (2.0)	0.1 0.04 0.66 0.08 0.27 0.3

*p* = probability value.

**Table 3:** Participants’ behavior regarding sharing prescription analgesics.

Questions/Answers	Urban (*N* = 500) *N* (%)	Rural (*N* = 500) *N* (%)	*p*
When you took prescription analgesic from another person, did you take also the accompanying package insert with information about a medication?			
Yes, verbal instructions Yes, written instructions No, nothing No answer	88 (18) 79 (16) 195 (39) 138 (28)	103 (21) 124 (25) 166 (33) 107 (21)	0.5 0.002 0.002
When you give prescription analgesic from another person did you give also the accompanying package insert with information about a medication?			
Yes, verbal instructions Yes, written instructions No, nothing No answer	99 (20) 66 (13) 167 (33) 168 (34)	129 (26) 111 (22) 153 (31) 107 (21)	0.39 0.009 0.002
Do you read paper-based instructions (package inserts) when you take someone’s prescription analgesic?			
Yes, always Usually Sometimes Rarely Never No answer	322 (64) 93 (19) 41 (8.2) 15 (3.0) 8 (1.6) 21 (4.2)	283 (57) 106 (21) 67 (13) 19 (3.8) 16 (3.2) 9 (1.8)	0.002 0.4 0.01 0.5 0.11
If you give to others and/or borrowed prescription drugs, did you inform your physician about it?			
Yes No No answer	32 (6.4) 310 (62) 158 (32)	58 (12) 325 (65) 117 (23)	0.02
Did your physician ever ask did you give and/or take from others analgesics that he/she prescribed to you?			
Yes No No answer	134 (27) 319 (64) 46 (9.2)	179 (36) 278 (56) 43 (8.6)	0.002

**Table 4:** Participants’ assessment of the likelihood of risk associated with sharing prescription analgesics.

Question	Urban		Rural	
	Mode (%)	M ± SD	Mode (%)	M ± SD
Could you harm yourself if you borrow a prescription analgesic from another person who is not a physician?	10 (32)	6.4 ± 3.3	10 (28)	6.0 ± 3.4
How likely is it that someone else who borrows a prescription analgesic from a person who is not a physician will harm themselves?	10 (31)	6.6 ± 3.1	10 (25)	6.0 ± 3.2
Do you think there are risks for you if you have lent your prescription analgesic to another person?	10 (31)	6.1 ± 3.4	10 (24)	5.6 ± 3.4
What do you think, what is the probability that you will develop side effects from prescription medications in general?	5 (25)	5.1 ± 2.8	5 (23)	5.1 ± 2.8
How likely is it that you will develop side effects after using prescription medications prescribed to someone else, if your problems are similar?	10 (22)	6.0 ± 3.1	5 (20)	5.6 ± 3.0
How likely is it that someone else will have side effects from the use of a prescription medication prescribed to you, even if they had a similar problem?	5 (20)	5.9 ± 3.0	5 (23)	5.6 ± 2.9

Acronym: M ± SD = mean ± standard deviation.

## Data Availability

The data presented in this study are available on request from the corresponding author. The data are not publicly available due to privacy reasons.

## Availability of Data and Materials

The datasets used during the current study with anonymous observations are available from the corresponding author upon request.
